# Sleep tight, play right: practical insights into sleep for soccer players

**DOI:** 10.3389/fspor.2026.1740420

**Published:** 2026-02-13

**Authors:** Christoforos D. Giannaki, Angelos Vlahoyiannis, Arnaldo L. Mortatti, Fabio Y. Nakamura, Gregory C. Bogdanis

**Affiliations:** 1 Department of Life Sciences, School of Life and Health Sciences, University of Nicosia, Nicosia, Cyprus; 2 Research Centre for Exercise and Nutrition (RECEN), University of Nicosia, Nicosia, Cyprus; 3 FSI Lab, Football Science Institute, Granada, Spain; 4 Department of Physical Education, Federal University of Rio Grande do Norte-UFRN, Natal, Brazil; 5 Research Center in Sports Sciences, Health Sciences and Human Development (CIDESD), University of Maia, Maia, Portugal; 6 Graduate Program in Physical Education, Federal University of Pernambuco, Recife, Brazil; 7 School of Physical Education and Sport Science, National and Kapodistrian University of Athens, Athens, Greece

**Keywords:** football, nutrition, performance, recovery, sleep hygiene

## Abstract

Over recent decades, interest in the relationship between sleep and athletic performance has grown substantially. Sleep is particularly crucial in soccer, where it significantly impacts recovery and performance, warranting careful consideration. Soccer players face several sport-specific challenges to optimal sleep, including frequent travel, competitive pressures, high training demands, late-night matches, exposure to artificial bright light, and early-morning training sessions. This narrative review aims to provide a comprehensive analysis of the literature on sleep’s influence on recovery, performance, health, and physical condition in soccer players, aiming to translate these findings into actionable, real-world strategies. Adequate sleep is a fundamental component of athletic performance and recovery. To address common sleep challenges in soccer, such as irregular competition schedules and frequent travel, it is essential to implement (i) systematic sleep monitoring and (ii) evidence-based interventions. Monitoring sleep presents challenges because it requires balancing practical feasibility with the level of detail needed to obtain meaningful insights into athletes’ sleep pattern. Effective intervention strategies include maintaining a consistent bedtime, strategically incorporating short naps, minimizing electronic device use before sleep, and adopting nutrition approaches that support recovery. By integrating these practices, coaches, players, and scientific staff can optimize both physical and mental readiness, ultimately enhancing performance and overall well-being.

## 1 Introduction

Recovery is an essential component of athletic training and competition, particularly in sports that combine intense physiological demands with the need for mental sharpness and cognitive performance (1, 2). Given the increasing frequency of matches and the reduced rest periods in modern soccer schedules, effective recovery strategies—such as low-intensity exercise, nutritional support, adequate hydration, psychological recovery, and sufficient sleep—are essential (3–7). Sleep is consistently rated by athletes and coaches as one of the most important recovery strategies and is therefore a central focus of this review (8).

The aim of this narrative review is to synthesize current knowledge on the critical role of sleep on performance, recovery and injury risk reduction in soccer players, updating a previous paper by Nedelec and colleagues (9). In addition, this review takes into account broader reviews (6, 10), which, while not focusing specifically on soccer, provide valuable practical applications for soccer players during intense training and competition thereby addressing existing gaps in the field. We focus on presenting a practical guide for improving sleep quality in soccer players, with evidence-based strategies that coaches, players, and sports scientists can implement. By providing actionable recommendations, this review seeks to enhance players' sleep hygiene and optimize both physical and mental performance in soccer.

This review first outlines the physiology and architecture of sleep, followed by an examination of sleep's role in the recovery processes of soccer players. It then discusses the typical sleep habits of soccer players and the impact of sleep on performance. Subsequently, the review addresses sport-specific challenges related to sleep, analyzing how characteristics inherent to soccer may disrupt sleep patterns and exploring potential strategies to mitigate these effects. Finally, the review presents practical recommendations in the form of a step-by-step sleep management strategy tailored for the periods before and after an important soccer match.

The literature for this review was identified through searches of the PubMed and Scopus databases. The search strategy was informed by the authors’ extensive research and practical experience in professional soccer and sleep science, using the keywords [“SOCCER”[Title/Abstract] OR “FOOTBALL”[Title/Abstract]] AND [“SLEEP”(Title/Abstract)]]. Selection was based on the relevance and quality of the studies, with a primary focus on peer-reviewed articles involving soccer players.

## 2 Sleep architecture

Among the various recovery strategies available to athletes, sleep has emerged as one of the most essential and accessible tools for optimizing recovery and performance. Sleep is essential for both physical and mental health. It alternates between two primary phases: Non-Rapid Eye Movement (NREM) and Rapid Eye Movement (REM) sleep (11). The NREM stage is divided into three gradual sleep depth levels: N1, N2 and N3, where stage N1 serves as the transition from wakefulness to sleep, typically lasting 1–5 min and accounting for about 5% of the sleep cycle. During the N1 sleep stage, muscle tone relaxes, and brain wave activity slows. Stage N2 of sleep (i.e., light sleep) accounts for approximately 50% of a sleep cycle, and is characterized by a further decrease in brain activity. This stage includes distinctive features such as sleep spindles and K-complexes. Sleep spindles are bursts of oscillatory brain activity visible on an EEG, occurring during non-rapid eye movement (NREM) sleep. K-complexes are large, slow waves that arise spontaneously or in response to external stimuli during NREM sleep) (12). These features reflect brief bursts of neural synchronization and cortical responses to external stimuli (11). These phenomena play a crucial role in memory consolidation and cognitive processing (13), facilitating the acquisition of new skills and tactics (14), which are not only essential for individual performance but also critical for understanding and executing team strategies in sports. The third sleep stage (N3), often referred to as “deep sleep” or “slow-wave sleep” (SWS), is essential for physical recovery. It facilitates the release of growth hormone, which is crucial for muscle repair and growth, and typically comprises about 20%–25% of a young adult's sleep, although it decreases with age (11, 13). The REM sleep stage typically begins approximately 70–90 min after sleep onset and is characterized by increased brain activity, vivid dreaming, and muscle atonia, which prevents most voluntary movements (13). This stage is vital for cognitive functions such as problem-solving and memory (13), which are essential for strategic thinking in soccer. Notably, there are very limited data on sleep architecture in soccer players, and this is something that future research should examine. To examine sleep architecture, techniques such as polysomnography are required, which unfortunately are not practical to implement in the world of soccer. In a recent study, sleep architecture was evaluated using wearable devices (WHOOP straps), and the results indicated no significant differences in sleep architecture parameters following home vs. away matches in elite soccer players (15). However, beyond sleep architecture, several sleep variables can be assessed more easily and are more practically available in soccer settings. These include sleep duration, defined as the cumulative time spent in physiological sleep states, and sleep regularity, reflecting the consistency of sleep–wake timing across days. Sleep efficiency, representing the proportion of time spent asleep relative to total time in bed, and sleep onset latency, defined as the elapsed time between bedtime and sleep onset, are also commonly assessed, along with perceived sleep quality. The topic of sleep assessment and monitoring in soccer players is further discussed in a dedicated section below.

## 3 The role of sleep in recovery

Sleep is a key component of recovery, supporting physiological and psychological restoration essential for peak performance (4, 6). Sleep promotes muscle recovery through protein synthesis and growth hormone release, and facilitates the consolidation of skills and memory, which are vital for tactical preparedness in soccer (16, 17). Additionally, sleep modulates mood and cognitive functions, crucial for maintaining focus and decision-making abilities during matches (18). Sleep also enhances immune function, helping athletes to withstand the demands of a rigorous soccer season (19).

Sleep structure, including the cyclical pattern of the sleep stages, plays a critical role in recovery (11). Disruptions in sleep architecture, such as those caused by irregular competition schedules, can impair recovery. Interruptions in deep sleep may reduce growth hormone secretion, hindering muscle repair (17), while disrupted REM sleep can affect cognition (i.e., attention and decision-making) (20) and visual reaction time (21), which are crucial components for maintaining mental clarity, alert state and tactical awareness (18).

During the deep stage of sleep (N3), particularly during SWS, the body undergoes significant physiological changes, including reduced heart rate, blood pressure, and respiratory rate, alongside increased blood flow to muscles, which facilitates the delivery of oxygen and nutrients necessary for muscle repair and growth, as well as the regeneration of body tissues (22). Moreover, the psychological benefits of sleep are equally important, as this period of rest can significantly reduce fatigue. This reduction is crucial for improving athletes' concentration and alertness, which are essential for executing complex tactical maneuvers and for reacting swiftly to dynamic game situations, and optimizing overall game performance (23).

An important aspect of sleep regarding the recovery process in athletes is the relationship between sleep and training load. Some studies reported a decrease in both sleep duration and sleep efficiency, as well as reduced immobile time (i.e., the total duration without detectable body movement during the time-in-bed interval), in athletes experiencing overreaching (24). It seems that changes in sleep patterns can be the result of non-functional overreaching and, on the other hand, may act as a contributor to the development of overreaching/overtraining in athletes (22). Therefore, systematic monitoring of both training load and sleep patterns is crucial.

## 4 Sleep assessment and monitoring in soccer players

There are various methods for evaluating sleep, both objectively and subjectively (Table 1). Objective methods include polysomnography, actigraphy, and nearable devices, while subjective methods involve questionnaires and sleep diaries (25, 26). In practice, in professional soccer, most often sleep is monitored through questionnaires within the general framework of wellness and fatigue assessment. Polysomnography, which is considered the gold standard for sleep evaluation, is not practical and is not commonly used in soccer players. However, in-home partial polysomnography devices are now available on the market, offering a valuable tool for a precise assessment of sleep architecture (i.e., scoring sleep stages) and identifying specific sleep disorders that could affect performance (27). Wearable sleep trackers offer a practical solution for sleep monitoring. Additionally, nearables—non-contact devices typically placed near the bed—offer an alternative approach to sleep monitoring using technologies like ballistocardiography. However, while wearables and nearables can validly estimate sleep duration, sleep onset latency, and wake after sleep onset, they are not capable of accurately measuring sleep architecture (i.e., sleep stages). At present, only EEG-based methods such as polysomnography can provide detailed insights into sleep staging. This distinction is critical for practitioners, as many commercial devices claim to “assess” rather “estimate” sleep stages, despite relying on indirect algorithms against PSG. Nevertheless, due to their ease of use, can provide valuable insights into sleep duration, efficiency, and disturbances, enabling tailored interventions (25). Recent studies have utilized wearable technology to monitor sleep, which has been instrumental in adjusting training and recovery programs (25). Notably, players sometimes react negatively to even actigraphs and wearables, feeling that wearing such devices continuously infringes on their personal life and increases their stress. Moreover, excessive sleep monitoring can lead to a behavioral phenomenon called orthosomnia, which can negatively interfere with the ability to sleep. Unlike insomnia, orthosomnia occurs when the individual becomes obsessive about trying to achieve “perfect sleep”, which can paradoxically impair adequate rest. This condition may result in some symptoms such as difficulty falling asleep at night (i.e., increased sleep onset latency), waking up during the night, waking up too early, non-restorative sleep, daytime sleepiness, mood swings, and problems with decision-making (28). To minimize the risk of orthosomnia, it is recommended to limit the frequency of sleep tracking (e.g., using devices only a few times per week rather than nightly). In addition, structured education on the interpretation of sleep data should be provided, emphasizing normal variability and prioritizing subjective sleep quality over minor fluctuations in tracker-derived metrics (29, 30).

**Table 1:** Summary of commonly used sleep assessment methods in soccer.

Method	Type	Main outputs	Validity (vs. PSG)	Practicality in soccer	Indicative cost
PSG	Objective	Sleep stages, macro-and micro-architecture, arousals, movement events and sleep disorders detection	Highest (reference standard)	Low (resource intensive; limited scalability)	High
Portable/in-home PSG or EEG-based systems	Objective	Sleep continuity and staging; disorder screening (depending on sensors)	High (system-dependent; generally closer to PSG than non-EEG devices)	Moderate (feasible for targeted assessments)	High
Actigraphy	Objective	Sleep timing, duration, regularity; estimates of sleep initiation and fragmentation	Moderate—high (for timing/duration)	High (field-suitable; longitudinal monitoring)	Moderate
Wearables/nearables (non-EEG)	Objective	Sleep timing/duration; awakenings/fragmentation	Variable (generally acceptable for timing/duration)	High (acceptability varies across players)	Low -moderate
Questionnaires (e.g., PSQI, ISI, ESS) and sleep diaries	Subjective	Perceived sleep quality, insomnia symptoms, sleepiness; contextual factors	Assess symptoms/perceptions	Very high (rapid, scalable; low burden)	Low

PSG, polysomnography; EEG, electroencephalography; PSQI, pittsburgh sleep quality index; ISI, insomnia severity index; ESS, epworth sleepiness scale.

## 5 Sleeping habits of soccer players

In a recent study involving 175 elite athletes, the participants reported that they needed 8.3 ± 0.9 h of sleep per night to feel adequately rested (31). However, these athletes averaged only 6.7 h of actual sleep, measured by actigraphy, with only about 3% of the athletes obtaining the sleep duration consistent with their stated need, while 71% fell short by more than 1 h (31). In that study, the subgroup of soccer players had an average sleep duration of 7.0 ± 0.7 h, and an average time in bed of approximately 8 h (habitual sleep onset at 23:39 ± 00:35 and habitual sleep offset at 07:45 ± 00:33) (31). These values are lower than the recommended sleep duration for the general population, and athletes may require even more sleep to recover from training and match loads. Moreover, in a recent systematic review where sleep was assessed exclusively through objective methods (i.e., using polysomnography and actigraphy), athletes were found to sleep an average of 7.2 ± 1.1 h per night, with a sleep efficiency (the ratio of total sleep time to time in bed) of 86.3% ± 6.8% (32). Soccer players also experience reduced total sleep time, longer sleep onset latency, and lower sleep efficiency on game days as well as during the weekly training microcycle, when compared to the recommended sleep duration for non-athletic populations (33). Reduced sleep duration negatively affects physical performance in soccer players. Reduced sleep time after night matches is generally associated with declines in performance and subjective well-being, including increased fatigue, stress, and muscle soreness (9). However, some studies have not reported these negative effects, suggesting that individual or contextual factors may moderate the impact (34). In addition, soccer athletes engaged in congested match schedules may experience greater mental fatigue and cognitive arousal (35), which can negatively affect sleep patterns, particularly during night matches (36). In non-soccer populations, sleep patterns appear to differ when comparing individuals from different continents (37). It is plausible that cultural norms, such as midday siestas in Southern Europe or shorter nocturnal sleep in East Asia, may shape athletes' sleep duration and timing, potentially affecting recovery patterns if soccer players from these regions adhere to such practices. Moreover, religious practices (e.g., Ramadan fasting), which are more prevalent in certain continents, can also impair sleep (38). To our knowledge, no study has directly compared the sleep patterns of athletes from European and non-European countries (e.g., South American countries). This remains an interesting topic for future research.

## 6 Sleep and performance in soccer players

In general, sleep loss in athletes has been shown to negatively affect several aspects of physical performance. According to a recent systematic review, the negative effects of acute sleep loss in athletes (not limited specifically to soccer players) are more pronounced in the afternoon than in the morning (39). Notably, these negative effects are observed only under total sleep deprivation and late sleep restriction protocols (i.e., waking earlier than normal) (39). Research highlights the link between poor sleep and soccer performance, although further studies are needed (40). Poor sleep quality is associated with slower reaction times, reduced decision-making accuracy, and increased injury risk in professional soccer players (40, 41). Moreover, partial sleep deprivation adversely affects anaerobic performance tests, muscle strength, and causes increased fatigue in the afternoon of the next day (42). However, performance in drill-based games may not be influenced by changes in sleep (43). While these associations are well-documented, specific direct causal links between sleep loss and injury risk cannot be ethically tested in experimental settings. As a result, most available evidence is correlational, emphasizing the need for high-quality longitudinal studies to further explore these relationships.

We should emphasize that there is limited evidence regarding the chronic effects of sleep loss and sleep restriction in soccer players (44). Theoretically, chronic sleep loss may lead to increased fatigue, impaired muscle recovery, reduced endurance and sprint capacity, and a diminished ability to perform high-intensity efforts—all of which are crucial for soccer performance. Additionally, cognitive functions such as decision-making and reaction time may be adversely affected, potentially resulting in more mistakes on the field (4). Collectively, these factors could negatively impact the overall performance of soccer players; however, it should be emphasized that the above considerations remain theoretical at this stage.

## 7 Specific challenges for soccer players

Intensive training schedules, high training loads (6, 32) evening matches, and frequent travels disrupt natural sleep patterns and circadian rhythms (45). Matches played in the evening can delay sleep onset and disrupt sleep architecture if not properly managed (4). Other factors that interfere with sleep include high social demands from social media, friends, sponsors, and others (6). Importantly, many causes of poor sleep in soccer players are difficult to control or change. For example, sport scientists and sleep specialists aiming to optimize sleep conditions cannot control fluctuations in training load and the scheduling of matches, which are typically determined by the League or the head coach. Establishing strong collaboration and mutual trust between the coach and the soccer team's staff is crucial to ensure that scientific insights are effectively implemented in practice.

Notably, sleep extension has been shown to improve reaction times, accuracy, and speed in athletes, highlighting its role in enhancing overall performance (46). Adequate sleep duration can also reduce perceptions of fatigue, enhance mood, and improve players' ability to cope with the physical demands of soccer (4). Additionally, sleep length appears to influence various aspects of cognition, which in turn affect soccer performance (4).

## 8 Sleep and the immune system

Numerous studies have demonstrated that inadequate sleep and sleep deprivation can impair immune function, increasing susceptibility to infections, including the common cold (47). A landmark study by Cohen et al. (48) found that individuals sleeping less than seven hours per night were almost three times more likely to develop an infection than those sleeping eight hours or more. Sleep is essential for optimal immune function, with SWS playing a key role in promoting adaptive immunity (19). As outlined by Besedovsky et al. (19), sleep fosters a hormonal environment that enhances T cell activation and supports the formation of immunological memory. Even short-term sleep deprivation disrupts these processes and increases levels of pro-inflammatory cytokines such as interleukin-1 (IL-1) and IL-12, and tumor necrosis factor –α (19).

Chronic sleep deprivation, defined as sustained restriction to ≤6 h per night over multiple nights, has more profound and systemic consequences in non-athletes, among which is persistent low-grade inflammation, characterized by elevated IL-6, TNF-α, and CRP levels (49). Furthermore, chronic sleep restriction promotes oxidative stress, endothelial dysfunction, and gut microbiota imbalance—factors that contribute to broader immunological disease risk and symptoms of overtraining (4, 50). In professional soccer, this vulnerability is heightened during congested fixture periods, where the high physical load compounded by poor sleep quality increases the incidence of upper respiratory tract infections (URTIs) (6, 51).

Together, these findings emphasize that both acute and chronic sleep loss significantly impair immune competence and may compromise resilience to infection, inflammation, and long-term health outcomes. Strategic interventions to promote optimal sleep duration and quality, such as consistent sleep routines and managing pre-sleep anxiety, can play a decisive role in accelerating recovery and maintaining peak performance and health in soccer players (52).

## 9 Sleep and muscle protein synthesis

The relationship between muscle mass and athletic performance is well documented. Muscle strength and endurance are critical for athletic performance (53), and their development is significantly influenced by mitochondrial and myofibrillar protein synthesis (17). Sleep is crucial for muscle recovery and growth, primarily through its role in protein synthesis. During sleep, the body enters a state of recovery where it restores and regenerates tissue, including muscle and mitochondrial proteins, thereby adapting to the exercise stimuli (54). Sleep deprivation can reduce the rate of protein synthesis and hinder recovery (17). This reduction is partly due to decreased secretion of growth hormone and changes in cortisol levels when sleep is inadequate. Studies have shown that protein ingestion before sleep can promote overnight muscle protein synthesis, improve recovery and promote adaptations to training (55, 56). This suggests that not only sleep quantity is important, but also that nutritional strategies before sleep are crucial.

## 10 Sleep challenges faced by soccer players

### 10.1 Schedule demands

Soccer players often face disruptions to their natural sleep patterns due to training, competition, and travel demands (6). Night matches can significantly delay sleep onset due to physical exertion and mental stress (cognitive arousal), coupled with the excitement and adrenaline (9). Teams in competitions like the UEFA Champions League, playing two to three matches per week, with one or more of these matches scheduled in the evening, often experience disrupted sleep routines (45).

### 10.2 Frequent travel and circadian disruption

Travel further complicates sleep management. Short-haul travel disrupts daily routines, while unfamiliar or uncomfortable hotel environments may impair sleep quality. The disruption of normal meal and sleep schedules due to travel itineraries can also contribute to reduced sleep quality (57). Additionally, the modern global nature of soccer requires teams to travel across continents for pre-season tours or competitive matches. The challenges are more severe with international travel due to jet lag and the need to cross multiple time zones. Jet lag disrupts the body's circadian rhythms, leading to significant sleep disturbances and impairing the synchronization of the body's internal clock with the new time zone, which can result in reduced performance (45). For example, European teams traveling to Asia or North America for pre-season competitions must adjust to significant time zone differences, which can disrupt their sleep patterns and negatively impact recovery processes. Adjusting to new time zones is particularly challenging when traveling eastward, as it requires a phase advance of the internal clock, which is more difficult to achieve than a phase delay (16). To mitigate these effects, teams can employ several strategies, including adjusting players' sleep schedules prior to travel (for example, scheduling slightly later wake-up times if a match is scheduled late at night) and selecting flights that minimize circadian disruption. Additional approaches include adjusting meal timing, planning training load and intensity before travel, and strategically using naps and caffeine when appropriate (i.e., after arrival). Finally, avoiding heavy meals before bedtime and using light-based interventions (e.g., bright light exposure in the morning) alongside melatonin may help to more effectively reset players' internal clocks (58).

Moreover, the cumulative effect of frequent travel, especially on long-haul flights, can lead to chronic sleep loss, which may not only impair performance but also increase the risk of illnesses as a result of a weakened immune system (4). As mentioned above, studies in non-athletic populations indicate that sleep can significantly suppress the immune system, thereby increasing the likelihood of these individuals becoming ill (19). It is reasonable to assume that if an athlete is ill, they may either miss training sessions or potentially have reduced performance in both competition and training, resulting in blunted adaptations.

### 10.3 Differences in sleep parameters between training and pre-match days, and different phases of the season

Sleep in soccer players differs across routine training days, match days, and late-evening training days. In particular, sleep patterns can vary significantly between training days and pre-match days, with pre-match anxiety often causing insomnia and reduced sleep quality (59). This variation highlights the need for specific sleep management strategies tailored to the needs of soccer players on different days (3). Moreover, it seems that the young soccer players exhibit longer sleep onset latency and reduced sleep efficiency (60). On match-days, bedtime is delayed and total sleep time and sleep efficiency appears to be reduced (61). Furthermore, on the night after the match, sleep duration is significantly shorter than on training nights, as shown in a study on female soccer players (62). Similarly, total sleep time is reported to be significantly lower the night after a match compared to both non-match days and training days in male soccer players, while it was longer in away compared to home matches (61). The time of training can also influence sleep. For instance, early time training seems to reduce sleep time and increase pre-training fatigue levels (63), while late-evening matches reduced subjective sleep quality, time in bed and total sleep time (36). Late-evening training also reduced total sleep time and increased sleep onset latency (64). Interestingly, the player's chronotype seems to influence the extent to which evening training affects sleep. In the study by Vitale and colleagues, sleep quality appeared to be poorer in players with a morning chronotype when training took place in the late evening (65).

Throughout the competitive season, athletes experience varying sleep patterns influenced by the intensity and content of their training and competition schedules. Research indicates that sleep quality and duration can fluctuate across different phases of the season, particularly during periods of heavy training loads (32). Mechanisms behind these disturbances include increased physiological arousal, muscle soreness, and maybe the psychological stress of impending competitions. Elevated stress hormones such as cortisol can disrupt circadian rhythms, making it harder to relax and transition into deep sleep stages (21). Furthermore, physical discomfort from intensified training can cause frequent awakenings and fragmented sleep patterns (32).

In contrast, during the tapering phase, when training load is reduced to prepare athletes for major competitions, improvements in sleep quality and duration are often observed (32). This reduction in stress allows the body to recover, leading to more restorative sleep. However, tapering is more common in individual sports, whereas in soccer, proper unloading before important competitions is rare, especially with congested fixtures. Nevertheless, studies involving young soccer players report longer sleep duration when training load increases (66).

Understanding these variations is crucial for developing effective training and recovery programs. Coaches and sports scientists should integrate targeted sleep interventions during preparation phases to mitigate the effects of heavy training loads. Strategies might include optimizing training schedules to allow more downtime, implementing relaxation techniques before bedtime, and ensuring conductive sleep environments.

## 11 Strategies for improving sleep in soccer players

In the context of enhancing sleep quality for soccer players, it is essential to differentiate between strategies that can be applied both after training sessions and match play. Optimizing post-training recovery routines is essential for enhancing sleep quality among soccer players. As elaborated below, effective recovery aids physical recuperation and prepares the body for restful sleep, which is essential for sports performance. Each set of circumstances presents unique challenges and opportunities for optimizing recovery through better sleep.

A gradual physical wind-down after training is vital. Engaging in low-intensity activities such as stretching, yoga, or light jogging can help transition the body from an active to a more relaxed state. These activities aid in reducing heart rate and muscle tension, setting the stage for a smoother transition to sleep (16).

### 11.1 Environmental behavioral adjustments

Creating a sleep-conducive environment in training facilities is another key strategy. This includes maintaining a quiet, dark, and cool environment in sleeping quarters. Sleep disturbances are more pronounced during periods of heavy training loads, such as the preparation period (32). During these times, ensuring an optimal sleep environment can help mitigate the negative effects of increased training demands and promote recovery and performance (3, 67).

Research involving young soccer players has demonstrated that taking a warm shower before bedtime can decrease sleep onset latency and enhance sleep efficiency. This improvement is likely attributed to thermoregulatory changes prompted by the warm shower, which facilitate the onset of sleep (68). A recent study showed that in the nights when the young soccer players slept alone in their rooms, they experienced shorter sleep onset latency, improved sleep efficiency, and increased total sleep duration than when they slept in a shared room during a soccer camp (69). These findings suggest a practical application that could enhance sleep quality in soccer players.

### 11.2 Sleep hygiene education in soccer players

Teaching good sleep hygiene practices can provide soccer players with strategies to improve sleep quality and duration, which are crucial for optimal performance and recovery. Expert guidance helps implement personalized sleep plans, tailored to their specific needs, leading to improved outcomes on the soccer field. A study by Fullagar et al. (67) found that acutely implementing a sleep hygiene strategy—such as maintaining a cool room temperature of approximately 17°C, using eye masks and ear plugs, and avoiding light or technological stimulation 15–30 min before bedtime—after a late-night soccer match led to longer sleep duration in amateur players. Similarly, an acute sleep hygiene program applied by Vitale et al. (70) revealed a reduction in the time to sleep onset after late-evening training sessions in soccer players. Notably, there is limited published data regarding the effectiveness of longer-term sleep hygiene interventions in soccer players. In a recent article, the implementation of a 4-week sleep hygiene program led to improved sleep quality, reduced sleep onset latency, and increased sleep duration. These data demonstrate the usefulness of acute and chronic sleep hygiene programs for improving sleep in soccer players (71).

Conducting workshops on hygiene practices is essential. These workshops should cover topics such as the optimal sleep environment, pre-sleep routines, and the impact of electronic devices on sleep quality. Interestingly, Höhn et al. (72) showed an impairment in deep sleep (N3) in young adults exposed to blue light-emitting screens, likely due to reduced melatonin levels (72). Involving a sleep medicine specialist is crucial, especially for players experiencing sleep disorders like restless legs syndrome. A specialist can provide targeted strategies and treatments, ensuring that sleep disturbances are properly addressed and do not hinder athletic performance.

### 11.3 Napping

A nap is a short sleep usually taken during the day. Naps can be particularly beneficial for athletes, providing extra restorative rest, especially when nighttime sleep is compromised. Napping can improve cognitive function, mood, and physical performance. A study involving soccer players following partial sleep loss showed that post-lunch napping led to improved performance in sprint tests and cognitive tasks (73). The timing of naps is crucial; ideally, naps should be taken early in the afternoon, to prevent interference with the onset of nighttime sleep. A nap duration of 20–30 min is recommended to avoid sleep inertia (74) which can impair cognitive function during the sleep-wake transition that occurs after a nap that reaches deep sleep (75). Moreover, even though long naps (i.e., 90 min) appear to enhance physical and cognitive performance (74), they may also lead to grogginess and increase the risk of sleep inertia, which in turn can negatively affect explosive performance, such as sprinting (76). In general, it appears that physical performance can remain enhanced for roughly 2–3 h after a nap (77, 78). Consequently, this approach is most applicable to training sessions, which are often scheduled in the afternoon, whereas it is harder to use before matches that take place in the evening. A recent systematic review by Lastella et al. reported that napping can have benefits especially in sleep restricted athletes (79). However, since there are studies indicating that napping cannot mitigate for the negative effects of sleep deprivation on soccer players' performance (42), it is more beneficial to focus on ensuring they get quality sleep at night rather than trying to counteract the effects with napping.

### 11.4 Nutritional interventions post-night matches

The role of nutrition in promoting restorative sleep is well documented (Table 2). Consuming a balanced diet, rich in nutrients like tryptophan, magnesium, potassium, and B vitamins, can enhance sleep quality (80). Implementing effective nutritional strategies for post-night matches is challenging but crucial (5, 81). While the timing of the post-workout evening meal is important, as consuming meals too close to bedtime might affect sleep, evidence remains mixed. Some studies suggest that late meals might disrupt sleep due to prolonged digestion (82, 83), but findings remain inconsistent (84, 85). Nonetheless, selecting foods that aid in the production of serotonin, such as those rich in tryptophan, may help in promoting sleep onset (16).

**Table 2:** Practical take-home nutrition strategies following night matches and in the pre-sleep period.

Recommendation	Scientific rationale/practical interpretation
Prioritize post-match carbohydrate intake to support glycogen resynthesis, and include protein to support muscle remodeling.	Soccer match-play induces substantial glycogen utilization; post-exercise carbohydrate supports replenishment, while protein supports overnight muscle protein synthesis and recovery processes.
If eating close to bedtime, avoid very large and high-fat meals; select lower-fat, lower-fibre, moderate-volume options.	The association between late eating and sleep is inconsistent across studies; however, large/high-fat meals may prolong gastric emptying and increase nocturnal gastrointestinal discomfort in susceptible individuals, potentially impairing sleep continuity.
When rapid sleep initiation is a priority, consider carbohydrate composition/timing as an individualized strategy (including higher-GI carbohydrate in some contexts).	Pre-sleep higher-GI carbohydrate has been associated with shorter sleep onset latency and improved sleep continuity in some studies, plausibly via effects on tryptophan availability and downstream serotonergic/melatonergic pathways; evidence is not uniformly athlete-specific and should be individualized.
Include tryptophan-containing protein foods as part of the late meal/snack when feasible.	Tryptophan is a precursor for serotonin and melatonin; food-based approaches may support sleep onset in some individuals without the risks of indiscriminate supplementation.
Micronutrients: prioritize adequacy (diet first); supplement only when deficiency risk is plausible or documented.	Magnesium and zinc have been linked to improvements in some sleep outcomes, but clinical trial evidence is limited/inconsistent and largely not derived from athletic cohorts; preventing deficiency is the most defensible strategy.
Functional foods (e.g., kiwi, tart cherry juice) may be trialed in training periods rather than introduced immediately before key matches.	These foods contain bioactives (e.g., antioxidants; melatonin/anthocyanins in tart cherry) and have shown improvements in sleep outcomes primarily in non-athlete samples; external validity to elite soccer is uncertain, supporting cautious, individualized implementation.
Post-match fluid and electrolyte replacement should be individualized; avoid excessive fluid boluses immediately pre-sleep.	Hydration strategies should aim to restore fluid and sodium losses while minimizing nocturnal awakenings due to urination. Evidence linking hydration status to sleep is mixed; nevertheless, individualized replacement is appropriate given high inter-individual variability in sweat losses and sodium requirements.
Use higher-complexity interventions (e.g., melatonin; multi-supplement protocols) under medical/qualified practitioner oversight.	Improper timing/dosing and inappropriate use can lead to reduced effectiveness or adverse effects; individualized application and monitoring are recommended.

Carbohydrates are essential for recovery after intense activity, especially in sports like soccer, where glycogen stores are depleted rapidly. Replenishing glycogen after night competition through adequate carbohydrate intake supports recovery and sleep. Both quantity and quality of carbohydrates consumed post-workout have been linked to sleep initiation, continuity, and overall sleep architecture (80). Incorporating carbohydrates with a high glycemic index can facilitate sleep onset by inducing a rapid postprandial glucose response, which probably promotes the synthesis and release of serotonin and its conversion into melatonin (85, 86). A pre-bed high-glycemic index meal has been reported to reduce sleep onset latency, increase sleep efficiency and prolong sleep duration (86). This enhancement in sleep duration was associated with faster visual reaction times the following morning, suggesting that the effect of glycemic index on cognitive performance may be mediated through improved sleep quality (86). This may have practical implications for athletes in sports like soccer, where quick visual reactions are essential for tracking the ball, anticipating opponents' actions, and making split-second decisions.

Micronutrients like magnesium and zinc may support post-competition nutrition by enhancing sleep quality. Magnesium improves sleep efficiency, sleep duration, and sleep onset latency (87), while zinc has been linked to improved sleep onset and maintenance (88). However, evidence from clinical trials remains limited and inconsistent, particularly within athletic populations. Hence, a well-balanced, nutrient-rich diet that prevents deficiencies is likely more practical and beneficial for overall sleep quality and recovery.

Recent studies have highlighted the potential benefits of certain foods and beverages, such as kiwi, tart cherry juice and tryptophan rich protein, in promoting better sleep quality (26). Kiwi, for instance, is rich in antioxidants and serotonin, which may have sleep-promoting properties. A study found that participants who consumed two kiwis one hour before bedtime over a four-week period reported falling asleep more quickly and improved overall sleep duration (89). Similarly, tart cherry juice has been shown to be beneficial for sleep due to its high content of melatonin and anthocyanins (90). Drinking tart cherry juice twice daily for two weeks has been shown to significantly increase total sleep time and sleep efficiency. These findings suggest that incorporating kiwi and tart cherry juice into the diet may be an effective strategy for enhancing sleep quality and recovery. However, since most of the studies were not conducted in athletes, the practical application remains lacking.

Despite the well-established evidence linking nutritional interventions with sleep, professional athletes often encounter challenges in implementing optimal nutritional strategies (26). This can be attributed to frequent travel schedules, which impede the ability to prepare sport-specific meals that meet the necessary nutrient and composition requirements (6, 26). Moreover, accommodation, such as hotels, often lack the necessary facilities for proper meal preparation. Even when players are at home, the influence of family-based routines and individual food preferences further complicates the consistent application of controlled nutritional practices aimed at optimizing sleep, recovery, and overall performance (26).

Hydration is another critical aspect of post-match recovery that can influence sleep. While the effects of dehydration on sleep are still controversial (91, 92), ensuring adequate rehydration after night matches is essential. However, fluid intake must be carefully managed to prevent excessive nocturnal awakenings that might disrupt sleep continuity. Equally important is maintaining appropriate sodium levels, as both excessive and insufficient sodium intake have been linked to sleep disturbances (93, 94). This highlights the need for individualized rehydration strategies and electrolyte balance, especially since sweat losses in soccer players can be substantial yet vary widely, even under the same exercise and environmental conditions (95). Moreover, many athletes do not fully replace fluid losses through voluntary intake alone, underscoring the importance of restoring electrolytes to normal physiological ranges without overshooting or undershooting requirements (95). By adopting tailored rehydration and sodium-replacement protocols, players can better safeguard sleep quality while optimizing overall recovery and performance.

Importantly, certain interventions, such as melatonin supplementation, intensive sleep monitoring, prolonged napping, and nutritional strategies, should be implemented with caution. Improper use may lead to unintended consequences or reduced effectiveness; therefore, these approaches should be individualized and applied under professional guidance.

In practice, post-night-match nutrition should prioritize a balanced, carbohydrate-containing meal alongside nutrient-dense foods and individualized hydration to support sleep and recovery. Flexible, evidence-informed strategies that account for real-world constraints and individual tolerance are likely more effective than reliance on isolated supplements.

### 11.5 Seeking medical assistance

When soccer players experience serious sleep problems or exhibit symptoms indicating sleep disorders, it is crucial to seek assistance from a doctor specialized in sleep medicine. Persistent sleep issues not only impair performance but also have long-term health consequences. Common sleep disorders that may affect athletes include insomnia, sleep apnea, and restless legs syndrome (RLS) (96). Sleep disorders are notably prevalent in elite soccer players, with evidence of high rates of poor sleep quality and subclinical insomnia. In one cohort of 111 players from the Qatar Stars League 68.5% of players scored ≥ 5 on the Pittsburgh Sleep Quality Index (PSQI), 27% reached levels of subthreshold insomnia on the Insomnia Severity Index (ISI ≥ 11), and 22.5% reported excessive daytime sleepiness (Epworth Sleepiness Scale > 8) (97). An exploratory study in 210 male professional soccer players demonstrated that insomnia severity was significantly associated with pre-sleep arousal, emotional factors, worry/rumination, and stimulant use, with arousal mediating these links (98).

A sleep medicine specialist can provide a comprehensive evaluation and accurate diagnosis, which is essential for effective treatment. These professionals are equipped to recommend behavioral strategies, prescribe appropriate medications, and suggest other therapeutic interventions tailored to the specific needs of the athlete. For instance, managing RLS might involve lifestyle changes, iron supplementation (if iron deficiency is a factor), or medication that targets dopamine pathways in the brain (99).

## 12 Practical application: a step-by-step sleep strategy before and after an important soccer match

**Figure 1:**
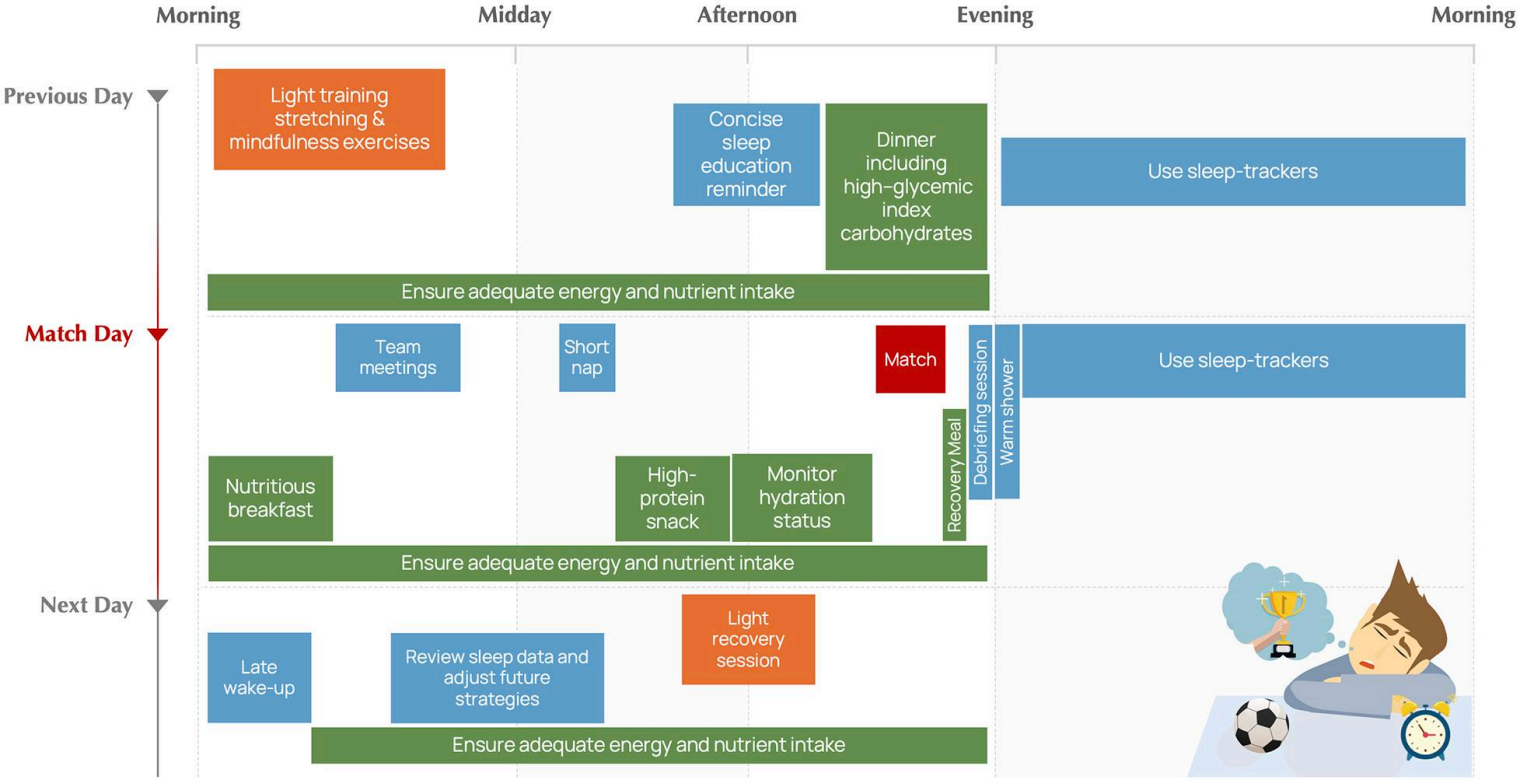
Typical post-night-match recovery and sleep-support strategy, which can be adapted according to the specific competitive, environmental, and individual athlete context.

In this section, we present strategies for managing sleep in a real-world context involving a professional soccer team preparing for an important evening match. These strategies are illustrated in graphical form in Figure 1 and are designed to help the team implement a comprehensive sleep strategy to optimize both performance and recovery.

### 12.1 Previous day

*Morning:* Players engage in a relatively low-load training session followed by a cool-down routine including stretching and mindfulness exercises to promote relaxation.

*Afternoon*: A concise sleep education reminder is delivered on the day before the match, aimed at reinforcing good sleep hygiene practices and stressing the importance of a restorative night’s sleep. Players are encouraged to consume a dinner rich in high-glycemic index carbohydrates to facilitate quicker sleep onset.

*Evening:* Players follow a pre-sleep routine that includes minimal use of electronic devices (avoiding engaging with stimulating material, such as social media, which can increase anxiety and delay sleep onset) and reading or light stretching instead. The goal is to be in bed between 10 and 11 PM, to ensure adequate sleep before the match day. Players are equipped with sleep trackers that allow monitoring of sleep quality while minimizing interference with natural sleep patterns.

### 12.2 Match day

*Morning:* Players have a nutritious breakfast and engage in team meetings. A short nap post-lunch is scheduled to ensure players are well-rested.

*Pre-Match:* High-protein snacks are provided 3 h before the match. Hydration levels are monitored.

*Post-Match:* Following the match, players participate in a debriefing session to mentally unwind from the match’s stress. Nutritionists provide meals that aid in recovery and are conducive to sleep.

*Evening:* Before bedtime, players take a warm shower to lower body temperature, a cue for the body to initiate sleep. Players wear sleep trackers that allow monitoring of sleep quality while minimizing interference with natural sleep patterns.

### 12.3 Following day

*Morning:* A late morning wake-up is scheduled to allow for extended sleep recovery.

*Afternoon:* Review of sleep data by a sleep specialist to adjust future strategies. A light recovery session is scheduled for the day.

## 13 Limitations

This review is narrative in nature and therefore carries inherent limitations. The selection of studies may introduce potential bias, and some recommendations are based on evidence from research that is not exclusively soccer-specific. Additionally, the absence of systematic methodology limits the ability to draw definitive conclusions. Furthermore, data from male and female players, youth and adult athletes, and different performance levels were sometimes pooled, which may reduce scientific precision given known differences in sleep physiology and recovery processes. Future work should aim to address these gaps through systematic reviews and experimental studies focused specifically on soccer populations.

## 14 Conclusion

This review highlighted the critical role of sleep in enhancing athletic performance and recovery for soccer players. Strategies such as creating a sleep-conducive environment, scheduling short naps at appropriate times, avoiding the use of electronic devices before sleep, tailoring nutrition, and using technology to monitor sleep are essential components of an effective sleep management program. Ensuring adequate, high-quality sleep will enhance players' performance, recovery, and well-being, ultimately leading to improved outcomes on the field. Looking ahead, future research should prioritize: (1) examining sleep architecture in soccer players using validated methods such as polysomnography to assess sleep architecture; (2) exploring cultural and environmental influences on sleep in non-European contexts; (3) conducting longitudinal studies on the relationship between sleep and injury risk; (4) developing and testing practical, evidence-based sleep hygiene interventions tailored for athletes; and (5) investigating the integration of sleep strategies with training and nutritional programs. These directions will provide a stronger evidence base for optimizing sleep and performance in soccer. From a practical perspective, integrating structured sleep strategies into daily training routines should be considered a core component of performance optimization in elite soccer.
